# Supplementary material for: Systemic Administration of Pegylated Arginase-1 Attenuates the Progression of Diabetic Retinopathy
Source: Cells. 2022 Sep 16;11(18):2890. doi: 10.3390/cells11182890 (PMC9497170; doi:10.3390/cells11182890)

Supplemental Figure S1. Evidence for the presence of PEG-Arg1 [>250 kD] in the neural retina of db/db mice. (A) Western blot of retinal expression of PEG-Arg1 [>250 kD] in db/db mouse treated with either PEG alone or PEG-Arg1. PEG-A1 expression is observed as a slightly diffuse band at >250 kD. **(B)** Frozen cross sections of the retinas at the head of the optic nerve were stained for PEG, CD31 (marker of endothelial cells) and DAPI (cell bodies).

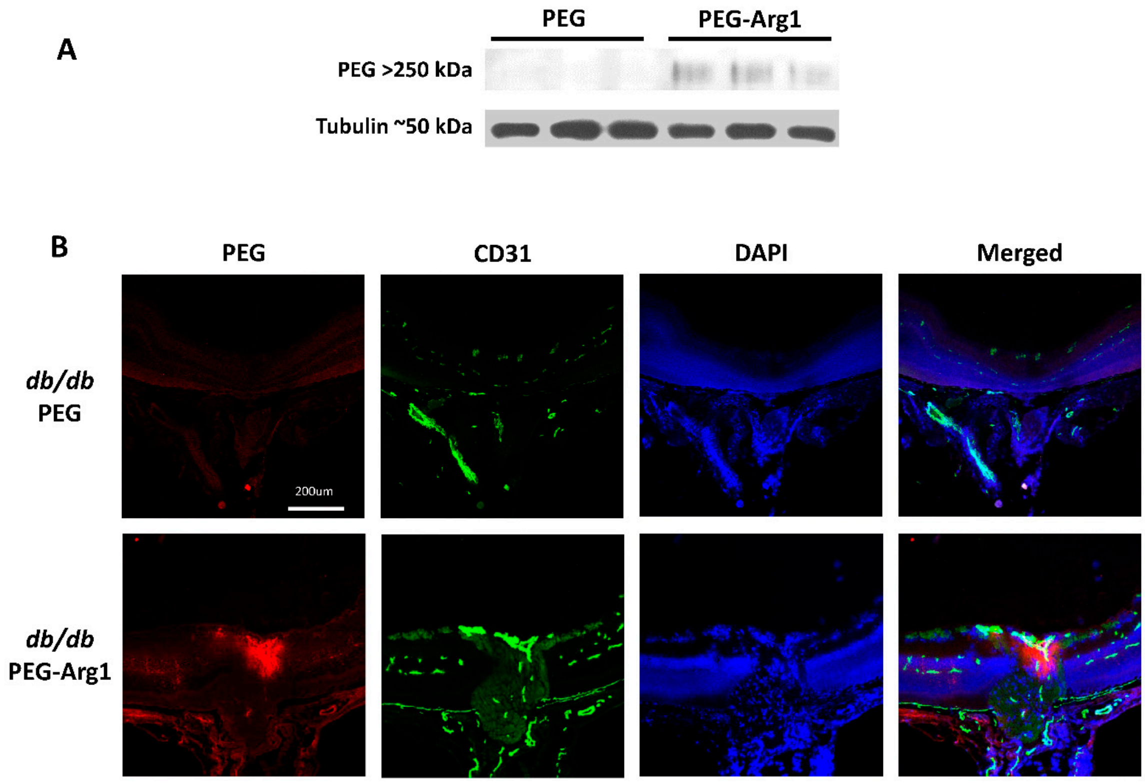

Supplement: Supplementary file 1 [file cells-11-02890-s001.zip › cells-1905022-supplementary.pdf]
